# What’s in a Name? Exploring the Nomenclature of Science Communication in the UK

**DOI:** 10.12688/f1000research.6858.2

**Published:** 2015-09-24

**Authors:** Sam Illingworth, James Redfern, Steve Millington, Sam Gray

**Affiliations:** 1 School of Research, Enterprise & Innovation, Manchester Metropolitan University, Manchester, UK; 2 School of Science & the Environment, Manchester Metropolitan University, Manchester, UK; 3 Research & Knowledge Exchange Office, Manchester Metropolitan University, Manchester, UK

**Keywords:** Science Communication, Public Engagement, Outreach, Widening Participation, Knowledge Exchange

## Abstract

This study, via a consideration of the literature, and a limited survey of active science communicators, presents concise and workable definitions for science outreach, public engagement, widening participation, and knowledge exchange, in a UK context.

Sixty-six per cent of participants agreed that their definitions of outreach, public engagement, and widening participation aligned with those of their colleagues, whilst 64% felt that their personal definitions matched those of their institute. However, closer inspection of the open-ended questions found the respondents often differed in the use of the nomenclature. In particular, the respondents found it difficult to define knowledge exchange in this context. It is hoped that this initial study will form the foundation of future work in this area, and that it will help to further develop the debate regarding the need for a consistent nomenclature across science communication.

## Introduction

Burns *et al.* (2003, pp. 183) define science communication as:

“…the use of appropriate skills, media, activities, and dialogue to produce one or more of the following personal responses to science (the AEIOU vowel analogy): Awareness, Enjoyment, Interest, Opinion-forming, and Understanding.”

This robust definition covers most aspects of communicating science to the public under a number of different guises. Where things start to get complicated is in the semantics regarding the different types of science communication, and in their appropriate use and classification.

Across UK institutions, science communication is often referred to using a variety of different terms, amongst them are science outreach, public engagement, widening participation, and/or knowledge exchange, but what do these terms actually mean? As well as institutional biases towards the ‘correct use’ of these terminologies there exists personal nuances in terms of their interpretation, which oftentimes depend upon the role of the person in question and how they perceive science communication to fit into their research and teaching practices, and beyond.

It can be argued that these definitions are simply a matter of semantics, but with science communication becoming more prevalent in grant applications and income generation (see e.g. the Research Council UK’s ‘Pathway to Impact’ report [ http://www.rcuk.ac.uk/ke/impacts/]), it is important for there to be consistency in what is a developing field. The advent of ‘Science 2.0’ (see e.g. Nattkemper, 2012) and what it entails is also an important driver behind having a clear and consistent nomenclature associated with science communication. Science 2.0 proposes a systemic change in the modus operandi of doing research and organising science, in which science communication will play a key part. With potentially large pots of money available in future grants, under specific terms and conditions, there needs to be a consistent terminology that can be drawn upon by the academic community and beyond.

According to the European Commission public consultation into Science 2.0 ( http://ec.europa.eu/research/consultations/science-2.0/background.pdf), something else that requires careful consideration is “the need to develop researcher and researcher reward schemes that reflect this (new) approach”. With potential reward schemes attributed to science communication activities, as well the creation of new job positions to fill these roles, it is important for all concerned to ensure that the language used in science communication is consistent.

This study, therefore, begins by discussing some of the definitions for science outreach, public engagement, widening participation, and knowledge exchange in the UK, derived from the common usage of these terms in the literature, and from the experiences of the authors. It then compares these definitions with the results of a survey of active science communicators from across the UK, and comments on the similarities and differences between the two, before identifying some suggestions for future nomenclature definitions within the field. The purpose of this study is to act as an initial scoping exercise, to begin to investigate and attempt to define a consistent set of nomenclature for use in science communication across the UK, and to act as a building block for further study and future debate.

## Literature analysis

The term ‘science outreach’ has been commonplace in research literature since the early 1990s, at which time the number of research articles on science communication started to increase. Many of these early articles describe science outreach as a school/education-linked activity, whereby academics are engaging with different groups of people such as the general public, students and teachers (see e.g. Greenler *et al.*, 1993; Kelter *et al.*, 1992). The term science outreach, which included activities such as mentoring, tutoring, giving presentations, supporting teachers and involvement with after-school clubs and summer schools, continued to become synonymous with school-related activity in to the 2000s, (e.g. Andrews *et al.*, 2005; Krasny, 2005). Recently, Ecklund *et al.* (2012) suggested that scientists involved in science outreach are often also engaged in some type of outreach involving school-aged children, demonstrating that the connection between school-related activity and science outreach remains strong.

Although much of the literature using the term ‘science outreach’ is based on work carried out in North America, this definition is similar to that used in the UK. Many organisations in the UK who fund science communication (e.g. Royal Society, Royal Society of Chemistry, Society of Biology, and the Wellcome Trust) use science outreach when explicitly discussing science communication with school children.

Although this link to school activity is present in the UK, there is some overlap with other commonly used science communication terms, in particular, public engagement; with some science communication practitioners using both terms together, e.g. schools outreach and public engagement.

In recent years there has been a shift from the deficit model of the ‘Public Understanding of Science’ towards a dialogue-based approach, which can be referred to as a ‘Public Engagement with Science and Technology’ ( Schäfer, 2008, and references therein).

Public engagement can be thought of as a way to restore public trust in science, by developing a two-way dialogue between the general public and the scientific community ( Wynne, 2006). Public engagement can foster global communication, enable shared experiences and methodology, standardize strategy, and generate shared viewpoints ( Cohen *et al.*, 2008). Furthermore, it can be defined as a deliberative process, promoted in both academic and policy circles, as a potential means to build public trust in risk decisions and decision-makers ( Petts, 2008). With regards to policy makers, public engagement can be viewed as both relevant and useful in a regulatory context (see e.g. O'Doherty & Hawkins, 2010), with the results of public discussions with scientists being a worthwhile process in scientific development ( Jones, 2007).

Recent years have seen increasing encouragement by research institutions and funding bodies for scientists to actively engage with the public, who ultimately finance their work ( Bowler *et al.*, 2012), and whilst many research institutions now have dedicated resources for public engagement activities, such activities are not yet considered essential ( Neresini & Bucchi, 2011). It is also unclear as to whether the institutional approach to public engagement is to focus on engaging with the public to promote their research and raise understanding, or if it is to open up a two-way dialogue in order to get their opinion on scientific research and protocol, especially in relation to potential political and ethical ‘hot potatoes’, e.g. geoengineering ( Parkhill & Pidgeon, 2011) and nanotechnology ( Jones, 2007).

The American National Centre for Media Engagement ( http://mediaengagedev.org/engagement/why-engage/difference-between-outreach-and-engagement) defines outreach as “a mechanism for delivering value-added content”, whereas engagement means, “collaboratively addressing community concerns.” This would seem to be consistent with the UK-centric arguments that have been laid out above, i.e. that outreach is a means of educating the general public (in particular school children), whereas public engagement involves a two-way dialogue in which the general public can offer advice and opinions as to the current state of scientific research. This approach to defining public engagement as something different from outreach is corroborated by Holliman *et al.* (2009, pp. 56) who state that:

“There is a heterogeneous community of practice operating in the space between what can be characterized as deﬁcit-informed ‘science outreach’—aimed primarily at increasing scientiﬁc literacy—and dialogue-informed ‘public engagement’ seeking to foster productive exchanges between scientists and other stakeholders (including members of the public).”

However, there still appears to be some uncertainty as to the difference between these approaches, and also to potential overlaps with regards to audiences; it is also unclear as to whether these definitions are consistent at an institutional level. As Rowe & Frewer (2005, pp. 251) remark:

“Imprecise definition of key terms in the ‘public participation’ domain have hindered the conduct of good research and militated against the development and implementation of effective participation practices.”

Concerns about “access” to high education began to emerge alongside the expansion of the university sector in the latter part of 19th Century, but a research agenda on differential access only began to emerge following the recommendations of the Robbins Committee in 1963 to expand university attendance ( Kettley, 2007). These concerns resurfaced in 1990s when the divide between universities and polytechnics ended, ultimately leading to a commitment by the 1997 Labour Government to again expand by the sector by tackling barriers to higher education. Consequently, Labour established the Office of Fair Access (OFFA).

Widening Participation involves interventions targeted at social groups under-represented in Higher Education (HE), in order to encourage them to attend university. According to the OFFA ( http://www.offa.org.uk/) this includes:

- Students from disadvantaged backgrounds
- Students with disabilities
- Students from some ethnic minority backgrounds
- Care leavers
- Part-time and mature students

With graduates benefitting from higher levels skills, knowledge, and access to the networks that are necessary to find higher paid work, the affordances of higher education are clear. Assuming disadvantaged social groups are afforded the same opportunities of access to employment through their university education, widening participation can help reduce social exclusion. It is not surprising, therefore, that the New Labour government largely reshaped the UK HE landscape in alignment with this ambition, with activity co-ordinated through Aimhigher, Lifelong Learning Networks, and the National Academy of Gifted and Talented Youth (see e.g. Frost, 2005).

However, the institutional landscape has since changed, with a greater onus now on the universities to independently deliver these objectives. In addition, university widening participation activity has come under greater scrutiny by the Higher Education Funding Council England (HEFCE), whereby universities opting to charge over £6k annual tuition fees, must also agree to Fair Access Agreements ( McCaig & Adnett, 2009).

In practice, widening participation aligns with the Pipeline or Learner Pathway model (see e.g. Clewell & Villegas, 1999); involving interventions designed to raise awareness and expectations of HE at various points within a learner’s education. With the emphasis on social mobility, widening participation focuses largely on targeting younger students from disadvantaged backgrounds utilising quantitative measures of poverty and deprivation, for example, the Index of Multiple Deprivation ( Deas *et al.*, 2003) and the eligibility for Free School Meals datasets.

In addition, widening participation can also be thought of as a consideration of the student lifecycle, beyond pre-entry and transition, to include university curriculum design, student support and employability. This follows concern that students from disadvantaged backgrounds perceive universities as ivory towers, i.e. places that are beyond their reach and are not for the likes of people like themselves (see e.g. Mangan *et al.*, 2010). It is important, therefore, to consider the impact of traditional university practices or institutional culture, not only on access, but also on the retention and progression of students from non-traditional backgrounds through HE.

Various conceptualisations of knowledge exchange have been in UK higher education discourse since the late 90s, when the Higher Education Reach Out to Business and Community (HEROBC) initially emerged. HEROBC was initially part of the so-called ‘third stream’ of funding, designed to sit alongside institutions’ teaching and research activities, and to provide funds for universities and colleges to pursue interactions with business and the wider community. At the time these interactions were exclusively centred on knowledge and/or technology transfer (rather than exchange), with the purpose of HEROBC being to develop the capacity and capability for knowledge transfer between Higher Education Institutions (HEIs) and other sectors. Typical activities that were funded through HEROBC included skills matching between university and business, and the provision of gateways to enable business to access university expertise and employability initiatives.

In 2001, HEROBC evolved into the Higher Education Innovation Fund (HEIF), which focussed on funding activities designed to increase the capability of universities “to respond to the needs of business, especially in instances that would lead to identifiable economic benefits” ( HEFCE, 2005, pp. 5) HEIF has since featured in four separate funding rounds, with explicit reference to knowledge exchange (rather than knowledge transfer) first emerging as a prominent part in December 2003 around the call for HEIF-2.

HEFCE, through the annual Higher Education Business and Community Interaction Survey (HEBCIS), now leads the categorisation of knowledge exchange activities. HEBCIS requires universities to report expenditure across various knowledge exchange categories including contract research, consultancy, CPD, business start-up, employability programmes etc. As university HEIF allocations are tied to levels of expenditure reported through HEBCIS, this exercise has been a big influence on what UK universities prioritise, resource and define in terms of knowledge exchange.

Despite the focus on expenditure, the important social, cultural and community role that universities play in wider society has not been entirely ignored. Influential voices have emerged around these concepts, most notably Professor David Watson (ex- Vice-Chancellor at Brighton University) who has been a champion of this societal agenda and the role that universities have to play within it, focusing on “civic and community” partnerships ( Watson, 2007).

Watson’s conceptualisation of knowledge exchange is rooted in a more engaged ‘two-way’ relationship between universities and external partners that sets out a much broader notion of knowledge transfer and knowledge exchange. John Goddard, the emeritus Professor of Regional Development Studies at Newcastle University UK, has also commented on the positions of universities as powerful engines of local and regional economic growth (see e.g. Goddard, 2009).

The most recent HEFCE definition states knowledge exchange “refers to HEIs’ engagement with businesses, public and third sector services, the community and wider public” ( http://www.hefce.ac.uk/glossary/#letterK). This adoption of a more explicit referencing of engagement within the knowledge exchange landscape has largely come about through a subtle yet important shift within funding council priorities prefaced, for example, within the Beacons for Public Engagement initiative (2008–2012) and leading towards the uptake of the impact agenda within the UK’s Research Excellence Framework (REF).

## Survey

In order to assess the current opinion relating to the definitions of outreach, public engagement, widening participation, and knowledge exchange in UK HEIs, a survey was conducted that asked participants to relate their understanding of science communication nomenclature.

The survey was conducted using Bristol Online Surveys ( https://www.survey.bris.ac.uk/), and comprised 8 questions delivered with a mixed-method approach (i.e. qualitative and quantitative questions). The focus was to evaluate the participant’s views on what constituted outreach, public engagement, widening participation, and knowledge exchange. It also aimed to assess whether or not the participants felt as though their own opinions aligned with those of colleagues and their institution. A copy of the questionnaire can be found in the supplementary materials section of the article.

Estimating the number of active science communicators in the UK is beyond the scope of this study. However, given that this study aimed to provide an initial scoping exercise into the thoughts and consistency of active science communicators across the UK, and taking into account the limited time frame and zero budget, an ideal sampling size of between 50 and 100 participants was chosen for the survey. Given the limitations in budget (which also precluded an interviewing/focus group approach), a convenience sampling strategy was adopted, in which the survey was advertised using the ‘psci-comm’ mailing list hosted by JISCMail, as well through the Twitter accounts of the authors, all of whom are active participants in UK science communication networks across the Twittersphere. The target audience were people that identified themselves as being active UK science communicators, which is why this sampling strategy was adopted. This study was carried out according to the British Educational Research Association’s (BERA) ethical guidelines for educational research, with all of the data in this study fully anonymised.

## Results & discussion

Answers to science communication questionnaire These are the responses to the questionnaire that was used in this study to assess practitioner’s definitions of nomenclature in relation to science communication. Click here for additional data file. Copyright: © 2015 Illingworth S et al. 2015 Data associated with the article are available under the terms of the Creative Commons Zero "No rights reserved" data waiver (CC0 1.0 Public domain dedication).

In total, 47 people participated in the survey during the allocated time frame of one month, and all bar one of them stated that they currently participated in outreach, public engagement, or widening participation events at their institute or company. Of the actively involved participants, 44 were located solely in UK, one in the Netherlands, and one was based in both the Netherlands and the UK. For the purposes of the analysis, only the 45 participants that were based in the UK, and who stated that they currently participated in science communication activities at their institute or company were selected. As well as being less than the intended sample size, it is acknowledged that the sample size of this survey is far too small to be able to make any generalisations about the nomenclature of science communication in a UK context. However, the responses will be able to help in the development of a potential framework, which can then be further discussed amongst the wider science communication community. It is also envisaged that this will help to foster the debate in terms of the importance of a standardised nomenclature for use across the UK science communication community and beyond. Given that the psi-comm mailing list contains several hundred active UK science communicators, and that between them the authors have several hundred Twitter followers that identify themselves as UK-based science communicators, it is disappointing that more people were not able to participate in the survey, but we believe that the number of responses is still sufficient for the purposes of this study.

**Figure 1.**
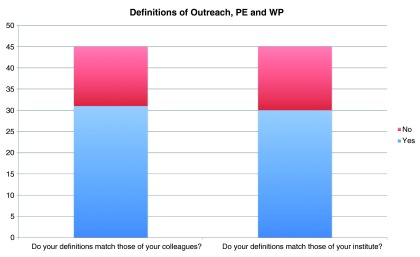
Stacked columns showing how participant’s personal definitions differed from those of their colleagues and institutes/companies.

Figure 1 shows the results of the survey in relation to h the participant’s personal definitions differed from those of their colleagues and their institutes.

Thirty-one of the forty-five participants (~69%) agreed that their definitions of outreach, public engagement, and widening participation aligned with those of their colleagues, whilst 30 (~67%) felt that their personal definitions matched those of their institute. Whilst the sample size is too small to draw any general conclusions about the consistency in science communication nomenclature across the UK, it is interesting to note that on the whole the majority of participants think that their definitions of outreach, widening participation, and public engagement match those of their colleagues and their institutes.

What is not clear from Figure 1 is if there actually is any agreement between the participants’ personal definitions of outreach, widening participation, and public engagement. As such, in addition to the questions regarding how the participants felt their definitions matched those of their colleagues and institutes, the survey also contained the following questions, which aimed to further explore how these different aspects of science communication are defined in the UK:

- How would you define outreach?
- How would you define public engagement?
- How would you define widening participation?
- How is knowledge exchange related to outreach, public engagement and widening participation?

**Figure 2.**
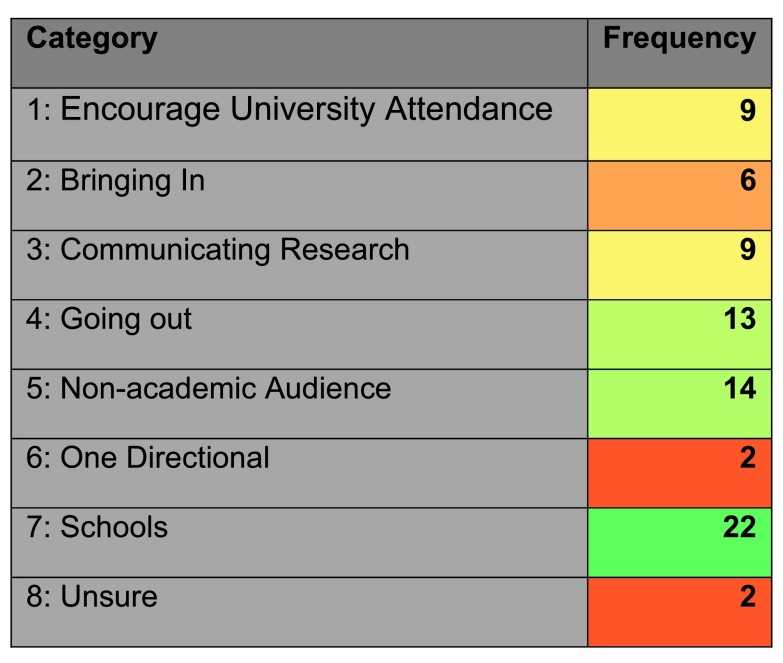
Major categories for ‘definitions of outreach’ and frequencies; listed in alphabetical order and colour coded according to frequency.

From the responses to these open-ended questions, the qualitative analysis tool NVivo was used to perform a qualitative thematic analysis. The different themes that were selected for each of the questions, along with the corresponding coding frequencies, are shown in Figure 2– Figure 5. To begin with, an open coding approach was taken, in which a number of major categories for each of the questions were deduced from the participant’s responses. These categories were then further investigated, including any potential overlaps. Following on from this initial open coding approach, the responses were re-examined in order to confirm that the major categories (and the concepts that these represented) were an accurate portrayal of the text. This stage was also used to explore how the categories and their concepts were potentially related. This methodology was carried out for each of the questions, and was carried out until descriptive saturation was reached, i.e. until there were no further codes, categories or themes found to be emerging from the analysis of the data.

From the thematic analysis and coding of the responses to the definition of outreach, there are eight major categories, which are displayed in Figure 2, the context for which are given below (there is some overlap between categories):

1. Encourage University attendance – These were responses that defined one reason for outreach as being that of an advertisement for encouraging participants to attend university, although not necessarily the university that was conducting the outreach activities.
2. Bringing In – These were responses that explicitly talked about bringing participants into the institute or research environment in order to conduct outreach activities.
3. Communicating Research – Responses that talked about the use of outreach as a way of advertising and communicating the research of the institute.
4. Going Out – These were responses that associated outreach activities as being those that took place outside of their institute.
5. Non-academic audience – Those responses that mentioned how outreach was for a general non-academic audience.
6. One-directional – These were responses that noted how outreach activities tend to be one-directional in their approach and delivery.
7. Schools – Responses that described how outreach activities were for school children and young people.
8. Unsure – Respondents who were unsure what was meant by the term outreach.

Regarding the responses to the definition of outreach ( Figure 2), what is immediately noticeable is that nearly half of the respondents made a direct association between science outreach and school education, and that schools and school children were their target audience. A smaller percentage (31%) of respondents, felt that such outreach was for non-academic audiences.

Thirteen respondents (~29%) felt that outreach takes place outside the university campus or research institute (in any location), whereas only six (~13%) noted the converse, i.e. that outreach activities are, and should be, carried out within the institute. One participant made the observation that:

“‘Outreach’ has negative connotations - it implies that the institution is doing all the work by ‘reaching out’ to an external group... I would define outreach as being the education-based programme of a large institution (such as a museum) where people are ‘brought in’ as opposed to coming as visitors of their own accord.”

Could it be therefore that the term outreach actually has negative implications for institutes should as museums, which require an influx of people to interact with their mainly on-site activities? Further probing would be needed to determine this hypothesis, wherein the participants’ responses would also need to be compared to their institutional roles.

Interestingly, some respondents explicitly discussed the connection between outreach and public engagement. One respondent stated that they believed outreach was “more educational than public engagement”, whilst two respondents believed outreach to be a subsection of public engagement, but did not elaborate on how the terms were differentiated in practice. Two of the participants made explicit reference to outreach being more of a one-way form of communication, with one of the participants noting that outreach is: “more one-way focused, having a scientist talk to a non-expert, not necessarily in a two-way conversation.”

In terms of the actual purpose of outreach, only sixteen (~36%) of the participants mentioned this explicitly, with nine (20%) of them stating that the purpose of outreach was to aspire audiences to pursue study in further education (although not necessarily at the institute conducting the research), and the same number of participants (two of the participants mentioned both of these categories as a rationale for conducting outreach) stated that the purpose of outreach was to communicate the research that was carried out by the institute.

Only two (~4%) of the respondents were unsure what was meant by outreach, with one of these participants stating that: “our organisation doesn't have an agreed definition of these terms.” Overall then, the sampling size is far too small to generalise in terms of absolute definitions, but from those that were surveyed, there appears to be quite a lot of disagreement in terms of the locale of where the outreach should take place, and what its ultimate purpose is, with more agreement on the target audience, i.e. non-academics with particular focus on school children and young people. This is in keeping with the literature review, which also discussed how many organisations in the UK who fund science communication use science outreach as the terminology when explicitly discussing science communication with school children.

**Figure 3.**
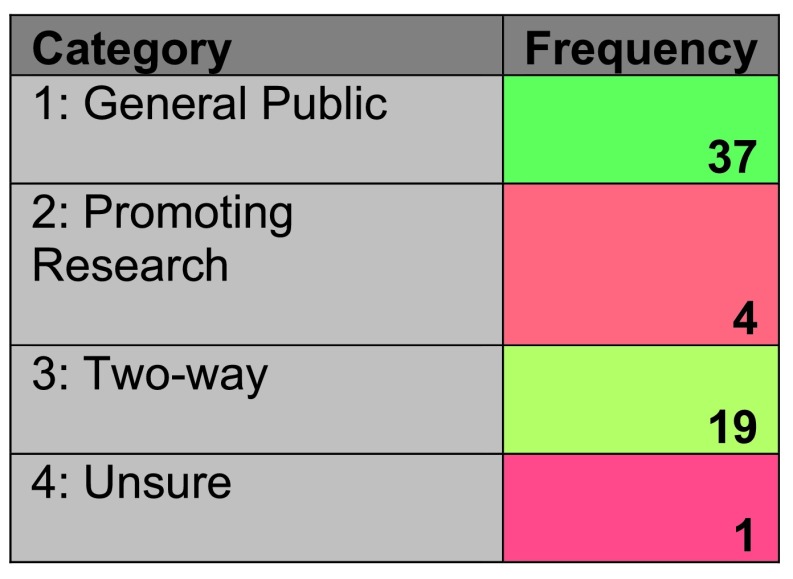
Major categories for ‘definitions of public engagement’ and frequencies; listed in alphabetical order and colour coded according to frequency.

From the coding of the responses to the definition of public engagement, there are four distinct categories, which are displayed in Figure 3, the context for which are given below (there is some overlap between categories):

1. General Public – Respondents who thought that public engagement was any activity that was aimed at the general public.
2. Promoting Research - Responses that talked about the use of public engagement as a way of promoting the research of the institute.
3. Two-Way - These were responses that noted how public engagement activities tend to be two-directional in their approach and delivery.
4. Unsure – Respondents who were unsure what was meant by the term public engagement.

From these responses, there were clearly two main themes: whom the public engagement was for, and what the purpose of the public engagement was. Some of the respondents talked about both (17), whereas a similar number talked about only the audience (21) and a smaller number (6) talked only about the purpose. In terms of the audience, the vast majority of the respondents that commented on the audience (37 out of 38) explicitly mentioned that public engagement should be for the general public. The remaining respondents noted that public engagement is for: “audiences not associated with schools and colleges,” which would seem to be in direct contrast to the audiences more readily associated with outreach. However, further questioning would be needed in order to ascertain if the other respondents also classified the general public as that which excludes school children. The whole notion of what constitutes the general public requires further investigation, as there was no clear set of categories as defined by the responses, with almost all of the respondents referring to simply “the public” or “the general public,” without going into details as to what was meant by that.

The respondents seem to mainly agree (19 out of 23) that the purpose of public engagement is to engage in a two-way dialogue with the public. Some of the sample responses that matched this definition included: “Public engagement can be a two-way process, with academics learning and incorporating feedback from the public”, “Public Engagement is ideally a two-way process, by which information is shared between two different groups”, and “Activities in which members of public audiences communicate with specialists in a way that has the potential to influence the specialists' activities.” The other responses regarding the purpose of public engagement (4 out of 23) were related to the promotion of the research and/or the institute itself.

Only one of the respondents was unsure what was meant by public engagement, with that participant stating that:

“I have no idea what the difference ( *between public engagement and outreach*) is supposed to be.”

From the responses of those that were surveyed, the most popular responses were that public engagement is for an audience of the general public, and that its purpose is to engage in a two-way discussion. Again, this is similar to the UK-centric view that was discussed in the literature review, that public engagement involves a two-way dialogue with the general public.

**Figure 4.**
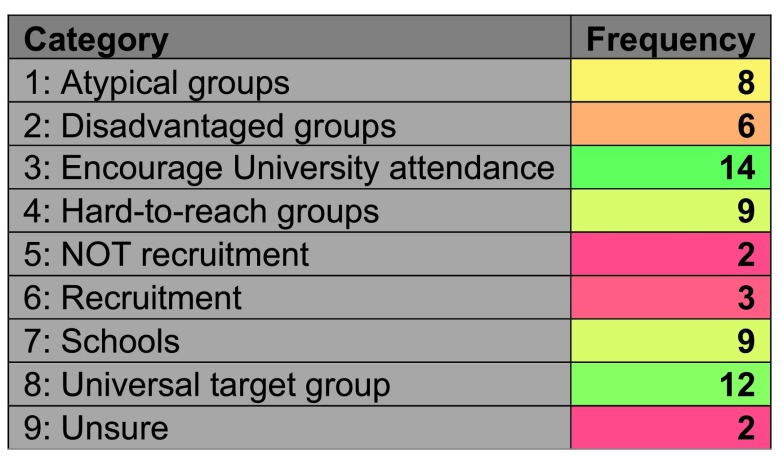
Major categories for ‘definitions of widening participation’ and frequencies; listed in alphabetical order and colour coded according to frequency.

From the coding of the responses to the definition of widening participation, there are nine distinct categories, which are displayed in Figure 4, the context for which are given below (there is some overlap between categories):

1. Atypical groups – Responses that mentioned the involvement of atypical groups.
2. Disadvantaged groups – Responses that mentioned the involvement of disadvantaged groups.
3. Encourage higher education – Respondents who thought that widening participation was any activity designed to encourage the participants to continue their studies into higher education.
4. Hard-to-reach groups - Responses that talked about the use of widening participation to interact with traditionally hard-to-reach groups.
5. Not recruitment - Respondents who felt that the purpose of widening participation was explicitly not as recruitment drive for their institute.
6. Recruitment – Respondents who felt that the purpose of widening participation was as recruitment drive for their institute.
7. Schools – Responses that talked about engaging with school children and young people.
8. Universal target group - Responses that talked a general universal target group.
9. Unsure – Respondents who were unsure what was meant by the term widening participation.

In terms of the purpose of widening participation, there was only one clear category, with 14 of the respondents (~31%) believing that it involved activities that encouraged students to continue their schooling into higher education. Within these responses, three of the participants explicitly stated that this involved a recruitment drive for the university, whereas two of the participants stated that they believed that it was *not* a recruitment drive. For future studies it would be interesting to investigate this dichotomy further.

The survey reveals less clarity, however, in terms of identifying specific target groups (some respondents referred to one or more target groups):

- Twelve respondents simply referred to a universal target group e.g. society, the public or simply engaging more people.
- Nine referred to groups that were under-represented or hard-to-reach, but most were unable to provide specific examples. Only one respondent, for example, referred specifically to minority ethnic status, only one to gender, and another referred specifically to disability.
- Nine understood widening participation in terms of relating specifically to schools or younger people
- Eight referred to atypical social groups, i.e. groups who would not normally or traditionally attend university
- Six referred to groups experiencing some form of disadvantage
- Three understood widening participation targets in terms of targeting geographical areas

A large number of responses refer to atypical social groups, people who express certain characteristics that appear to be defined against some notion of what constitutes a normal student, for example “those with different cultural attitudes and ideals.” This finding chimes with the concern that university staff continue to understand diversity in a way that reproduces the notion of universities as places for some normalized subject, defined against an atypical Other. Only one respondent, for instance, referred to widening participation in terms of curriculum support/design for widening participation of students already in HE.

Two respondents claimed to have not heard of the term widening participation before, whereas two expressed a rather cynical perception that widening participation had become “hijacked by university recruitment agendas”, and another respondent referred to widening participation as “the annoying habit of targeting minority groups.”

The number of respondents was too small to generalise in terms of absolute definitions, but from those that were surveyed, it appears that the purpose of widening participation is to aspire students to continue their education into higher education, which is in keeping with the definitions discussed in the literature review. Exactly which audiences should and are being targeted is less obvious from the responses, and any future study should look to further investigate why this is the case, and to what extent it is determined by institutional protocol and/or personal preference.

**Figure 5.**
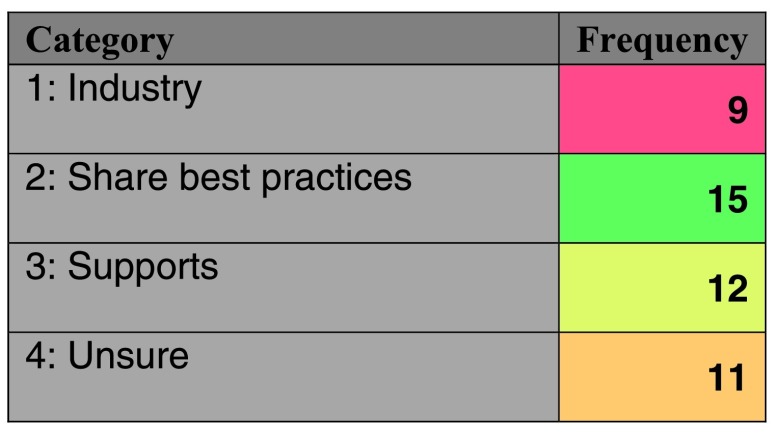
Major categories for ‘how knowledge exchange is related to outreach, public engagement and widening participation?’ and frequencies; listed in alphabetical order and colour coded according to frequency.

From the coding of the responses to the definition of knowledge exchange, there are four distinct categories, which are displayed in Figure 5, the context for which are given below (there is some overlap between categories):

1. Industry – Respondents that mentioned the involvement of industry.
2. Share best practices – Respondents who thought that knowledge exchange involved the sharing of best practices.
3. Support - Responses that talked about how knowledge exchange involved the support of outreach, public engagement, and/or widening participation activities.
4. Unsure - Respondents who were unsure how knowledge exchange is related to outreach, public engagement and widening participation.

What is immediately clear from Figure 5 is that there is no consistent definition or understanding of knowledge exchange amongst respondents. There are instead four major categories to the responses, which represent a reasonably broad range of concepts, definitions and views (or not) of knowledge exchange in evidence.

One of the most popular responses (~27%) was that knowledge exchange exists to support outreach, public engagement, and/or widening participation activities. The responses coded in this category ranged from simple expressions such as “it aids it”, to more verbose explanations, including:

“Knowledge exchange is an important part of all of the above, however they all require a lot more than just passing on knowledge, you have to also pass on your enthusiasm and do this in an enjoyable and engaging manner, to achieve good Outreach, PE or WP.”

This idea of exchanging information and expertise is related to the most popular response to this question (~33%), which was that knowledge exchange is a way of sharing best practices, be it between the expert and the audience in a two-way dialogue (in a similar manner to public engagement), or because it:

“Allows external stakeholders to influence our activities but also allows us to share expertise with or influence them.”

Nine of the respondents (20%) identified knowledge exchange as related to interactions between universities and industry, leading to increased economic activities. Given the nature of knowledge exchange that was laid out in the literature review, it was perhaps surprising to see that only one of the respondents explicitly mentioned HEIF, noting that:

“For the first time under the last HEIF round universities were asked to provide Knowledge Exchange Strategies to indicate how they would allocate their HEIF resource.”

Eleven of the respondents (~24%) were unsure of how knowledge exchange was related to outreach, public engagement and widening participation. Either because they had “never come across the term 'knowledge exchange'”, or because they were unsure how the terms were all related. Again, whilst the sampling size of this survey was too small to make any definite statements, of all the questions that were posed by this survey, it was the issue of knowledge exchange, which was responsible for the largest amount of misunderstanding.

## Conclusion

Perhaps the most noticeable result from this study is that the open-ended responses to the survey resulted in a wide range of definitions of outreach, public engagement, widening participation and knowledge exchange amongst the participants, despite the quantitative data indicating that the majority of the respondents felt that their definitions of outreach, knowledge exchange, and public engagement agreed with those of their colleagues. This would seem to indicate that further communication is required both within and between institutes to ensure a level of consistency amongst science communicators, although this specific question will require further study.

The lack of demographic data means that it is not possible to comment in terms of the different roles within the UK science communication community. However, based on the current literature, and the results of this study, the following broad definitions are offered for each of the four considered topics:

**Outreach:** a one-way discourse, in which scientists communicate their research to the general public, with particular focus on school children and young people.

**Public Engagement**: a two-way dialogue, in which scientists converse with members of the general public in a mutually beneficial manner.

**Widening Participation:** any activity that engages with social groups under-represented in HE, in order to encourage them to attend university.

**Knowledge Exchange:** any activity that involves engagement with businesses, public and third sector services, the community and the wider public, which involves the sharing of best practice, and which can be monitored for funding purposes.

It is acknowledged that there is still some overlap between these definitions, for example a school assembly given by a university researcher at a local school might well be classed as being an outreach, widening participation, and knowledge exchange activity. In such instances it is important to consider the context of these classifications. In this example, the researcher’s faculty might classify the activity as outreach, the university’s widening participation team (or equivalent) may catalogue it as a widening participation activity, and the knowledge exchange offices (or equivalent) could acknowledge it in their records for HEFCE.

It is important for science communicators to consider the context in which their activity takes place, because depending on its classification, the activity may be eligible for different amounts of funding from different areas of resource. This consideration of context is especially important when applying for external funding, where science communicators will be expected to outline the specific area(s) in which their activity can be categorised. These activities are also extremely important in terms of determining the pathways to impact of future REFs, and whilst widening participation tends to align with teaching outcomes, rather than research, it should be acknowledged that important to note that widening participation will likely become part of the Teaching Excellence Framework – an outcome based model that the UK government proposes to evaluate quality of teaching.

The results of the survey also indicate that the respondents were less comfortable defining terminology around knowledge exchange than they were about outreach, public engagement and widening participation. The job titles and functions of respondents may be an important factor here, and further work is needed to confirm this. A future study is planned which also aims to assess how the different perceptions of science communication nomenclature would break down according to stakeholders. For example, the ways in which an academic, museum and learned society view these definitions might be very different. An international study, with a much larger target audience, is also required so as to assess differences in perceptions of the science communication lexicon between countries, both those traditionally associated with the field and those that are not. This study should also present participants with an open-ended question to define any further terms within the science communication vernacular which they believe to be important, and why.

This study, via a consideration of the literature, and a survey of science communicators, has presented concise and workable definitions for outreach, public engagement, widening participation and knowledge exchange. However, as with all names it is important that the people using them feel comfortable with them, and also that there is at least some form of consistency within the field (and beyond) as to their usage. This consistency will only come about by communication both within and between institutions, and this study aims to act as a starting point for such conversations, with planned future work aiming to further explore the perceptions of science communication and its nomenclature amongst a much wider target audience.

## Data availability

The data referenced by this article are under copyright with the following copyright statement: Copyright: © 2015 Illingworth S et al.

Data associated with the article are available under the terms of the Creative Commons Zero "No rights reserved" data waiver (CC0 1.0 Public domain dedication). 

F1000Research: Dataset 1. Answers to science communication questionnaire, 10.5256/f1000research.6858.d97179 ( Illingworth *et al.*, 2015).

## Supplementary Material

**Science communication questionnaire.**

This is the questionnaire that was used in this study to assess practitioner’s definitions of nomenclature in relation to science communication.
