# Supplementary material for: What’s in a Name? Exploring the Nomenclature of Science Communication in the UK
Source: F1000Res. 2015 Sep 24;4:409. Originally published 2015 Jul 28. [Version 2] doi: 10.12688/f1000research.6858.2 (PMC4582756; doi:10.12688/f1000research.6858.2)
Supplement: Supplementary file 2 [file f1000research-4-7640-s0001.tgz › e0b9a095-8062-4c44-b713-dc3954f5ec7c.pdf]

Questionnaire on Outreach, PE & WP

1. Do you participate in any science Outreach, Public Engagement (PE) or Widening Participation (WP) events at your institute/company?

2. In which country is your institute/company located?

3. Does your personal definition of Outreach, PE and WP match those of your colleagues?

4. Does your personal definition of Outreach, PE and WP match those of your institute/company?

5. How would you define Outreach?

6. How would you define PE?

7. How would you define WP?

8. How is Knowledge Exchange (KE) related to Outreach, PE and WP?

9. Any other comments?
