# Supplementary material for: What’s in a Name? Exploring the Nomenclature of Science Communication in the UK
Source: F1000Res. 2015 Sep 24;4:409. Originally published 2015 Jul 28. [Version 2] doi: 10.12688/f1000research.6858.2 (PMC4582756; doi:10.12688/f1000research.6858.2)
Supplement: Peer review history

# Peer review: Referee response for version 2

**DOI:** 10.5256/f1000research.7640.r10466

**Published:** 2015-10-07

**Authors:** Cornelia Lawson

**Affiliations:** 1 Centre for Business Research, University of Cambridge, Cambridge, UK

The authors have provided a thorough review addressing all reviewer comments. The new version provides a much clearer analysis and robust conclusions. Shortcomings are addressed and I very much hope the authors will be able to further pursue their research to answer some of the questions that have remained.

I have read this submission. I believe that I have an appropriate level of expertise to confirm that it is of an acceptable scientific standard.

# Peer review: Referee response for version 2

**DOI:** 10.5256/f1000research.7640.r10465

**Published:** 2015-09-29

**Authors:** Sarah R. Davies

**Affiliations:** 1 Department of Media, Cognition, and Communication, University of Copenhagen, Copenhagen, Denmark

The authors have thoroughly responded to the reviewers' comments, undertaking a more robust analysis of the data and more nuanced reflections on the both the data and the wider literature. As a result it's a much stronger and more interesting paper. I very much enjoyed the opportunity to engage with it.

I have read this submission. I believe that I have an appropriate level of expertise to confirm that it is of an acceptable scientific standard.

# Peer review: Referee response for version 1

**DOI:** 10.5256/f1000research.7380.r9682

**Published:** 2015-08-19

**Authors:** Cornelia Lawson

**Affiliations:** 1 Centre for Business Research, University of Cambridge, Cambridge, UK

The article looks at a series of, perhaps, interchangeable terms used in the context of science communication and knowledge exchange and how they are understood by those working in academia. It provides an informative and brief literature review and concise definitions for each of the terms: outreach, public engagement, widening participation and knowledge exchange, and presents results of an original survey of science communicators.

The research question is important and timely as concepts of science communication and knowledge exchange merge and the impact agenda gains in importance in the UK and elsewhere. However, I have some mayor concerns regarding the empirical analysis. The sampling framework and the sample are not sufficiently described making it difficult to interpret the results. In particular, at several points the authors mention science communicators but do not specify whether these were the target population. They also make no estimate as to how many people were reached and which sectors they work in. It is therefore not clear whether the final sample can be considered representative (as is claimed in making use of the phrase 'fair cross-section').

The sample is very small and one is left wondering whether interviews with a similar number of respondents would not have enabled a better analysis and insights. The final sample also includes employees of universities and of companies but it is not clear how they are distributed and whether answers differ between the two. This is of importance as some of your conclusions seem to assume a homogeneous institutional environment, i.e an academic work environment. More over, we do not know whether respondents are academics, administrators, or professional science communicators. No collecting such information is a major flaw.

The use of word clouds to analyse your data strikes me as very odd. The word clouds add no information what so ever and I would suggest removing them. First of all because it can only highlight word frequency, which it does not do very accurately (e.g. it does not look for word stems), and because it does not allow to reader to know the actual frequency or importance of words but only their relative frequency and importance. Other packages such as NVivo should be used as it allows to identify relationships between words and to deduce linkages between the different answers provided.

Alternatively a comparison of definitions found in the literature and definitions provided by respondents would provide more interesting results and allow some important insights into how concepts are diffusing.

A few final remarks on the literature review: The article does not specify when public engagement and widening participation were introduced as concepts and/or policies in the UK and I think this could be added. The impact agenda within the UK's REF is mentioned towards the end of the review and from there it seems that outreach, public engagement and knowledge exchange are part of this agenda, while widening participation is not. Perhaps this could be commented on here and in the conclusion.

I have read this submission. I believe that I have an appropriate level of expertise to confirm that it is of an acceptable scientific standard, however I have significant reservations, as outlined above.

# Response

**Published:** 2015-09-14

**Authors:** Samuel Illingworth

**Affiliations:** Manchester Metropolitan University, UK

Thank you for your helpful comments, which we have taken on board, and which have been used to improve the overall quality of the paper. 

Regarding the sampling framework and the sample size, we agree that this was not discussed in enough depth, but as discussed in the response to the previous comments this has now been rectified and discussed in the main body of the paper. Any claims that the paper makes in terms of generalisations have also been re-addressed, as we agree that given the sample size this is not appropriate.

We also agree that not collecting further demographic data in terms of job titles and roles was an oversight, and as discussed in the responses to the previous comments, and also in the paper itself, this is something that we intend to do in future studies, using the findings of this study as a starting point for further investigation. 

We accept that the use of word clouds to illustrate the key points in the data was at best inconclusive and at worst potentially misleading. However, this has now been addressed, with a more in-depth analysis using NVivo now provided, which allows for a more considered examination in terms of the relative frequency and importance of the responses. 

Regarding the introduction of these terminologies in terms of policies, concerns about “access” to high education began to emerge alongside the expansion of the university sector in the latter part of 19th Century, but a research agenda on differential access only began to emerge following the recommendations of the Robbins Committee in 1963 to expand university attendance. These concerns resurfaced in 1990s when the divide between universities and polytechnics ended, ultimately leading to a commitment by the 1997 Labour Government to again expand by the sector by tackling barriers to higher education. Consequently, Labour established OFFA. This has now been commented on in the literature survey. 

In terms of the relationship between widening participation and REF, widening participation tends to align with teaching outcomes, rather than research. However, it is accepted that there is a blurring of the boundaries, e.g. a research-focused public engagement event may well inspire someone to attend university who wouldn't have otherwise. In addition to this, it is important to note that widening participation will likely become part of the Teaching Excellence Framework – an outcome based model that the UK government proposes to evaluate quality of teaching. This has also been commented on in the conclusions, as suggested.

# Peer review: Referee response for version 1

**DOI:** 10.5256/f1000research.7380.r9685

**Published:** 2015-08-17

**Authors:** Sarah R. Davies

**Affiliations:** 1 Department of Media, Cognition, and Communication, University of Copenhagen, Copenhagen, Denmark

This is an interesting topic, and one that is ripe for study. As the authors point out, there is a growing amount of activity in and funding for various kinds of science communication (they point to the phenomenon of 'Science 2.0', but they may also like to think about this relates to scholarship on Mode 2 knowledge production - e.g. see Etzkowitz & Leydesdorff (2000)). There is definitely lots going on, a mass of different terms for related activities, and a degree of slippage between different terminologies.

As the authors themselves point out, though, there are some problems with their methodology, and I also feel that the analysis and discussion would benefit from a fuller engagement with the literature. I'll take the latter point first.

To start with, I think the authors need to more clearly make a case for their focus on the terms that their analysis is based on (that's rather clumsily put - but I hope my point will become clear). Their starting point, unreferenced, is that "Science communication is often analogously and interchangeably referred to as science outreach, public engagement, widening participation, and/or knowledge exchange". Yes - but many other terms are also used, including dialogue, PUS, scientific literacy, tech transfer, third stream activity, and co-enquiry. Why were *these* terms selected (and indeed why not 'science communication' itself)? This sense of arbitrariness is heightened by a literature analysis that bounces around in a somewhat haphazard way. The authors ignore, for instance, work that has attempted to define some of these terms (Burns *et al. *(2003), whom they cite at the very start of the paper, offer an extended discussion of terms they see as similarly related to science communication, including scientific literacy and PUS; the work of the NCCPE - http://www.publicengagement.ac.uk - is also a valuable resource for seeing how different forms of science communication have been framed). By interpreting the 'participation' that the science communication literature often refers to (e.g. in the Rowe & Frewer article they cite) as being about 'widening participation' initiatives they also make a bridge to an area of university outreach and external relations that is infrequently discussed in the main body of science communication research (wrongly so, I think - but that's another discussion); rather, the term 'participation' tends to be used in the tradition of deliberative theory and public participation in science research and policy (a randomly chosen example would be Bogner (2012)). All this is to say that things like 'public engagement' and 'widening participation' actually have rather different histories, and different communities around them - so it doesn't surprise me, for instance, that some respondents hadn't heard of widening participation initiatives. These are likely to be *organised* quite different within university systems. I was also surprised that the authors didn't spend more time considering other empirical work on practitioner definitions of these terms or of science communication generally. Some of my work has treated this (not that it's necessarily essential reading - but see, e.g., Davies (2013), but Jason Chilvers, John C Besley, and Kevin Burchell, among others, have also published analyses of how scientists and other practitioners of public communication tend to define and understand it (e.g. Chilvers (2008); also Besley (2010) ). 

So - in short, I think both the framing of the study, and the analysis and discussion, would benefit from a more thorough engagement with the qualitative literature that has built up around the meaning and practice of science communication. I also have some comments about the methodology and analysis - largely to do with the need for more explanation as to what the former was, and why it was chosen. For instance, the authors note that the survey was "advertised via email, social media accounts, and the ‘psci-comm’ mailing list"; earlier, they say that the study aimed to understand opinion on definitions of the four terms "in UK HEIs". What, exactly, is the target participant group? Everyone working in UK HEIs? A certain subset of this population, those who are interested in science communication? The HEIs themselves, as institutions (i.e. as organisations with particular brands)? How was the sampling strategy (the distribution of the survey) designed to reach the desired population? In terms of the survey advertisement via "email and social media accounts": email to whom, and why, and which social media accounts (and why - but you get the picture...)? The PSCI-COMM list is distributed to a large group of those interested or working in or researching science communication, in the UK but also internationally. Is the target population therefore 'science communicators'? (In practice almost all respondents had participated in science communication in some way, so perhaps so. But this needs to be clear.) [Apologies - I've just re-read your comment in the conclusion that respondents only being active science communicators is a weakness of the study. But in that case, if you're interested in everyone working in UK HEIs from chancellors to cleaners, you need to justify why you used a survey methodology, and why you thought your sampling strategy would reach everyone.]

I would also like to know more about how the analysis, and particularly the qualitative analysis, was carried out. Using word clouds is a rather basic means of analysis, as it tells us only how often a word is cited, not the context in which it is used or the meaning that is attached to it; discussion of the qualitative responses is therefore important. Were these coded in some way? How were themes robustly identified? The data evidently did not reach saturation (to use a term from grounded theory), as the authors emphasise the diversity in the definitions given. Do you think you needed a larger sample size, or is there so much interpretative flexibility in these terms that saturation would never be reached?

The authors do note, in the conclusion, that the sample size is a weakness of the study, and I would agree with this. As far as I can tell you were also not able to identify the role or situation of your respondents, only whether they had participated in science communication activities in the past. This, to me, also weakens the results significantly - or at least represents a lost opportunity. There are big differences between different kinds of communities within universities and as participants in communication (e.g. scientists, outreach officers, admin staff, PR teams, tech transfer offices...). It seems unlikely that these communities would have homogeneous definitions of the terms in question - or even have heard of them all (as your findings suggest).

I want to close - and I do apologise for the lengthy review - with a broader point. The issues that you touch upon raise some fascinating questions. I would love to know more, for instance, about the relations between individuals and teams in universities working on 'widening participation' and on public engagement-type projects, and I would be very curious to know if academic staff give different kinds of definitions of these terms to those who organise science communication on a professional basis. In this regard I would encourage you to look beyond finding the 'right', or even commonly used, definitions of particular terms. Alan Irwin has talked about 'third order' studies of science communication, which explore how different terms are mobilised by different groups, and the kinds of effects that this has (see Irwin (2008) In: Bucchi M and Trench B (eds), *Handbook of Public Communication of Science and Technology*, London and New York: Routledge). Your study suggests interesting ways to explore how very different meanings can be applied to the same terms - it would be great to hear more, in the future, about how these different meanings are made to matter in particular contexts (such as, to go back to the very beginning, moves towards Mode 2 and entrepreneurial universities). I wish you all the best with this future research.

I have read this submission. I believe that I have an appropriate level of expertise to confirm that it is of an acceptable scientific standard, however I have significant reservations, as outlined above.

# Response

**Published:** 2015-09-14

**Authors:** Samuel Illingworth

**Affiliations:** Manchester Metropolitan University, UK

Thank you for your comments, which we found extremely useful, and which have helped us to reshape the paper into a more effective and considered study. 

The comment regarding the choice of outreach, public engagement, widening participation and knowledge exchange, and why these were chosen for selection is a pertinent one. However, we believe that in the context of UK institutions, these are the phrases that are most readily used in relation to science communication. PUS and scientific literacy are terms that are, in our experience, used more in an American and European context, whereas tech transfer and third stream activity would arguably be covered by knowledge exchange. However, we agree that the wording in the text was a bit strong, and has now been changed. We have also made a note in the conclusions regarding that future studies should also present participants with an open-ended question to define any further terms within the science communication lexicon which they believe to be important, and why. 

Regarding the bounciness of the literature analysis, we think that the literature that we have engaged with illustrates that there is no concise definition of the terms that we have chosen, and as such serves its purpose as a useful introduction and rationale for this study. It is understood that the selected terms have different historical and academic contexts, but we believe that the literature that we have engaged with illustrates this point.

As noted in the response to the previous reviewer’s comments, we have now addressed the issue of the sampling size and the sampling strategy, and accept that not doing so in the previous version of this paper was an oversight. It should have also been made more apparent that only the responses from UK-based participants were included in the analysis, and this has now been corrected for in the text. In addition to this, only the participants who reported as being active in science communication were included in the analysis; again this has now been made clearer in the text. 

We agree that Wordle was not the most academically rigorous way of presenting our data, and that word frequencies could be potentially misleading. As such the word clouds have been replaced with tables that outline the major themes for the thematic analysis, and the frequencies associated with them. A far more detailed description of how this thematic analysis was carried out using NVivo is also given. Through an open coding approach, descriptive saturation was reached, and this is also now discussed in detail in the text. 

Again, we acknowledge that the lack of sufficient demographic data reduces the potential depth of the analysis, representing a lost opportunity that we hope to address in future studies. This will include, as noted in the conclusions, investigating how different stakeholders define different aspects of science communication, and how and why this changes depending on job title, and both across and between institutes. 

Thank you for your suggestions regarding future studies, especially your comments regarding how definitions of the nomenclature may change depending on job role. This has now been further discussed in the conclusions where we talk about future work, where with a broader study and more demographic data such questions will be targeted. We agree that there may well be no ‘right’ definition of particular terms, but having broad and workable descriptions might well help better communication between the different practitioners of science communication.

# Peer review: Referee response for version 1

**DOI:** 10.5256/f1000research.7380.r9683

**Published:** 2015-08-10

**Authors:** Paige Brown Jarreau

**Affiliations:** 1 Manship School of Mass Communications, Louisiana State University, Baton Rouge, LA, USA

**Summary: A re-analysis of the data and a significant re-write is advised.**

The authors have provided a fine if brief literature overview of definitions of science outreach, public engagement, widening participation and knowledge exchange. This is also a timely topic, as the need for evidence-based science communication has grown in today's complex ecosystem of science new media and outreach efforts on the part of scientific institutions.

However, there are major flaws in this paper as it is currently written and presented. The sample is extremely small for an online survey - this sample would have been more appropriate for in-depth interviews with each respondent. This is acceptable as long as the audience/population is clearly defined. Are those who answered the survey mostly active scientists, or mostly dedicated science communicators? This could make a big difference to participants’ definitions of science communication, outreach, public engagement, widening participation and knowledge exchange.

Why only 47 respondents? The ‘psci-comm’ mailing list serves a much larger number of people than this (and the authors also don’t make any estimate of how many people they reached with their survey.) How long was data collected for? The authors should more clearly spell out their sampling goals and procedures. 

“These results suggest that in a still emergent field, the participants of this survey are likely to be the driving influence behind the definition of science communication at an institutional level.” How so? Please make this statement, and how/why this conclusion was reached, more clear.

“If the participants that took part in this survey represent a fair cross-section of people working in science outreach, public engagement, and widening participation across the UK then it is somewhat alarming that such a significant proportion of them feel as though the fundamental basis on which their work is founded lacks such clemency in its definitions.” The authors should be VERY careful in making any statement regarding the implications of this survey representing a fair cross-section of people working in science outreach, etc., as per my comments on the sampling procedure above. The authors don’t present any data on the basic demographics of the respondents. This was a critical oversight. 

In the analysis of survey responses, I am not under the impression that Wordle is an academically rigorous method for mapping word and concept frequency. Other qualitative analysis tools such as AtlasTi would have been preferable, even if figures were created in Wordle for demonstration purposes only. Wordle images, while a visually appealing, make it very difficult to quickly gauge relative word frequency. 

The authors’ methodology in qualitatively analyzing, coding and interpreting the survey responses is very unclear, if not missing altogether. How were the survey responses approached during data analysis? Were the authors’ primarily looking for responses that corresponded with how the scientific literature defines concepts of outreach, engagement, etc., or were novel or conflicting definitions also coded and analyzed (e.g. open vs closed coding?)? From the description of survey response analysis, it is not clear if the data analysis reached saturation, or what theory or framework guided the textual analysis.

It would be very useful to know the demographic information of those respondents who provided alternative definitions of outreach and engagement, and of those respondents who were unable to define widening participation, etc. Are these scientists? Are these professional science communicators? This type of basic information would enrich the meaning of these results. The authors state only in their conclusion section that the respondents were “active science communicators.” What type of science communicators? How many were involved in academic institutions? I think this type of information is paramount to the authors’ interpretation of their findings, and the lack of this data is a significant weakness in this paper.

What insights did we glean from the authors’ survey of 47 science communicators regarding the definitions and terminology surrounding science outreach, public engagement, widening participation and knowledge exchange beyond what the authors presented in their literature review? What insights to the field and definition of science communication were uniquely contributed by this study? In this the authors are also unclear. It would have been useful for the authors to compare and contrast at greater length their respondents’ definitions of these concepts to published scientific literature definitions.

I have read this submission. I believe that I have an appropriate level of expertise to confirm that it is of an acceptable scientific standard, however I have significant reservations, as outlined above.

# Response

**Published:** 2015-09-14

**Authors:** Samuel Illingworth

**Affiliations:** Manchester Metropolitan University, UK

Thank you for your comments, which were very insightful, and which we have used to address these flaws, thereby helping to improve the academic rigour of the paper. 

Estimating the number of active science communicators in the UK is beyond the scope of this study. However, given that this study aims to provide an initial scoping exercise into the thoughts and consistency of active science communicators across the UK, and taking into account the limited time frame and zero budget of this study, an ideal sampling size of between 50 and 100 participants was chosen for the survey. Given the limitations in budget (which also precluded a more in-depth interviewing / focus group approach), a convenience sampling strategy was adopted, in which the survey was advertised using the ‘psci-comm’ mailing list hosted by JISCMail, as well through the Twitter accounts of the authors, all of whom are active participants in science communication networks across the Twittersphere. The target audience were people that identified themselves as being active UK science communicators, which is why this particular mailing list was adopted. Given that the psi-comm mailing list contains several hundred active science communicators, and that between them the authors have several hundred Twitter followers that identify themselves as UK-based science communicators, it is disappointing that more people were not able to participate in the survey, but we believe that the number of responses is still sufficient for the purposes of this study. This has now been made clearer in the text. 

Regarding the two statements relating to science communication at an institutional level and the cross-sections of science communicators that were represented, we agree that it was inappropriate to make such generalisations, and so these have been removed from the text. 

We agree that Wordle was not the most academically rigorous way of presenting our data, and that word frequencies could be potentially misleading. As such the word clouds have been replaced with tables that outline the major themes for the thematic analysis, and the frequencies associated with them. A far more detailed description of how this thematic analysis was carried out using NVivo is also given. Through an open coding approach, descriptive saturation was reached, and this is also now discussed in detail in the text. 

In terms of the lack of demographic data that was collected in regards to this survey, we realise that this was an oversight, and we have commented on this in the text. However, we still believe that this study is worthwhile, and that it has presented a relatively concise set of definitions that can be used for further discussions.

# Peer review: Referee response for version 1

**DOI:** 10.5256/f1000research.7380.r9684

**Published:** 2015-08-06

**Authors:** Laura Fogg Rogers

**Affiliations:** 1 Science Communication Unit, University of the West of England, Bristol, UK

This article attempts to define common definitions for inter-changeable terms used in science communication through a literature review and survey of science communicators. It is a timely article, as this is a much sustained debate in both academic and practitioner circles, and relates to discussion in the literature about funding, incentivising and rewarding science communication. More particularly, it links directly to efforts to define the ‘impact’ of science communication through processes such as the Research Excellence Framework and the Public Engagement with Research agenda. The article is well grounded in this literature and refers to many of the current debates in this field.

The article uses survey results to move forward the debate on these definitions, attempting to find consensus amongst science communication practitioners. However there were only 47 participants involved in the survey, which is a low sample size. The authors acknowledge this point however, and also note that it may be useful to repeat the survey with researchers and professionals who don’t consider themselves science communicators.

Despite these limitations, the survey results indicate that while 66% of respondents state that their definitions match those of colleagues, the resulting qualitative data indicates wide differentiation. These results are presented through Wordle diagrams. While these are visually interesting, further qualitative thematic analysis, along with matrix analyses for coding frequency, could also have added to the results. Percentages are given for word frequency, but a methodology for how this was obtained would be useful.

The resulting concluding definitions are a useful addition to the field, but still produce considerable overlap between activities. The article notes that further work is needed, and I would concur that this is a topic ripe for more in depth research.

I have read this submission. I believe that I have an appropriate level of expertise to confirm that it is of an acceptable scientific standard.

# Response

**Published:** 2015-09-14

**Authors:** Samuel Illingworth

**Affiliations:** Manchester Metropolitan University, UK

Thank you for your comments, which are greatly appreciated, and which we have used to improve both the content and presentation of our study. 

As you have pointed out, we acknowledge that the number of participants in this survey is small, and the purpose of this study was to act as an initial scoping exercise amongst science communicators to try and determine how they would summarise the terms of outreach, public engagement, widening participation and knowledge exchange within the umbrella term of ‘science communication.’ The idea for this paper was to begin to further develop the conversation that needs to take place in regards to how science communicators use the terminology in their fields, initially on a UK-wide basis. 

Given that limited demographic data was taken in this study (which was in hindsight an oversight), the Wordle diagrams were simply a tool to indicate the frequency with which certain words occurred. However, it was not at all clear from the original paper how this had been done, and further thematic analysis has now been carried out using NVivo, with the text being rectified to reflect this. The word clouds have also been replaced with tables that outline the major themes for the thematic analysis, and the frequencies associated with them.
